# Chloroplast genome of the invasive *Pyrus calleryana* underscores the high molecular diversity of the species

**DOI:** 10.1007/s13353-022-00699-8

**Published:** 2022-05-06

**Authors:** Marcin Nowicki, Matthew L. Huff, Margaret E. Staton, Robert N. Trigiano

**Affiliations:** Department of Entomology and Plant Pathology, University of Tennessee, Knoxville, TN USA

**Keywords:** Callery pear, Plastome, Reference-guided assembly, Plastid genome annotation, Pears

## Abstract

The complete chloroplast genome of *Pyrus calleryana* (GenBank OM541581.1) was developed by de novo assembly from whole-genome sequencing data. Reference-guided (*P. phaeocarpa*) read mapping and assembly were followed by annotation and phylogenetic comparisons. The 159,965 bp *P. calleryana* chloroplast genome represented 36.56% GC content with a classical quadripartite architecture and two inverted repeats regions (IRs; each 26,392 bp) separating the large single-copy region (LSC; 87,942 bp) and the small single-copy region (SSC; 19.239 bp). In total, 125 unique features were annotated in that genome, including 83 protein coding genes, 38 tRNA coding genes, and 4 rRNA coding genes. Phylogenetic analyses based on the whole chloroplast genome sequences placed the *P. calleryana* among other Rosaceae plants, specifically among the Asian species of *Pyrus*.

**Supplementary Information:**

The online version contains supplementary material available at 10.1007/s13353-022-00699-8.

## Introduction

Callery pear (*Pyrus calleryana* Decne.) is species of pears native to China, Korea, Japan, and Taiwan (Sapkota et al. 2021) and belongs to the Oriental origin of the genus. This diploid pear species shows the number of chromosomes typical for *Pyrus* with 2C = 34 = 588Mbp (Sapkota et al. 2021). Its economic importance derives from its hardiness and pest and environmental stress tolerance; this renders it an important rootstock for *Pyrus* spp. and beyond (Culley and Hardiman 2007; Santamour and Demuth 1980). Resistance to *Erwinia amylovora* Burrill Winslow (fire blight) led to import of *P. calleryana* to the USA in early twentieth century, to be grafted with edible pear scions (Culley and Hardiman 2007). Owing to attractive ornamental features, many commercial cultivars of *P. calleryana* have been released to great commercial success (Culley and Hardiman 2007; Santamour and Demuth 1980). Soon thereafter the species has escaped cultivation and is now declared invasive in an increasing number of US states (Sapkota et al. 2021; Culley and Hardiman 2007); the mechanisms behind the invasiveness include hybridizations with other *Pyrus* species flowering at the same time, and high mutation and migration rates (Sapkota et al. 2022).

Genomics resources for Callery pear would enable the species detection using molecular methods as well as insights into genetic basis of traits of interest, invasiveness, and better understanding of how the rootstocks influence the scion traits in fruit production. In this study, we developed the complete chloroplast genome for the invasive *P. calleryana*, using the reference-driven read mapping, assembly, and annotation.

## Materials and methods

As the first step towards our research goal, we re-used the available whole-genome sequencing data of Callery pear accession from China (Jinshan Fruit Tree Test Station in Shanghai Academy of Agricultural Sciences; GenBank SRR16505594) towards reference-guided assembly of chloroplast genome. The original sample collectors deposited voucher specimens in their laboratory. This short-read archive was previously used to mine microsatellite markers for phylogenetic comparisons with other *Pyrus* species (Jiang et al. 2019; CRR019697 of China National Center for Bioinformation; reads trimmed and filtered for high quality). The overall genome coverage was 13.8 × and all details pertaining to DNA isolation, sequencing, and quality assessment were published (Jiang et al. 2019). Other related datasets of *P. calleryana* were used for critical evaluation of our bioinformatics approach (SRR7135497; SRR7135498; SRR7135500). Exclusively the trimmed clean reads of high quality were used for the analyses.

The largest available *Pyrus* chloroplast genome (*P.phaeocarpa*; MK488091.1) was used as reference for short-read mapping by BWA-MEM2 v.2.2.1 (Vasimuddin et al. 2019). Successfully mapped reads from SRR16505594 and others were assembled using pilon v.1.23 (Walker et al. 2014) with that same reference. Annotation using GeSeq v.2.03 from the Chlorobox suite (Tillich et al. 2017) followed. The code used is available as Suppl. File 1.

**Fig. 1:**
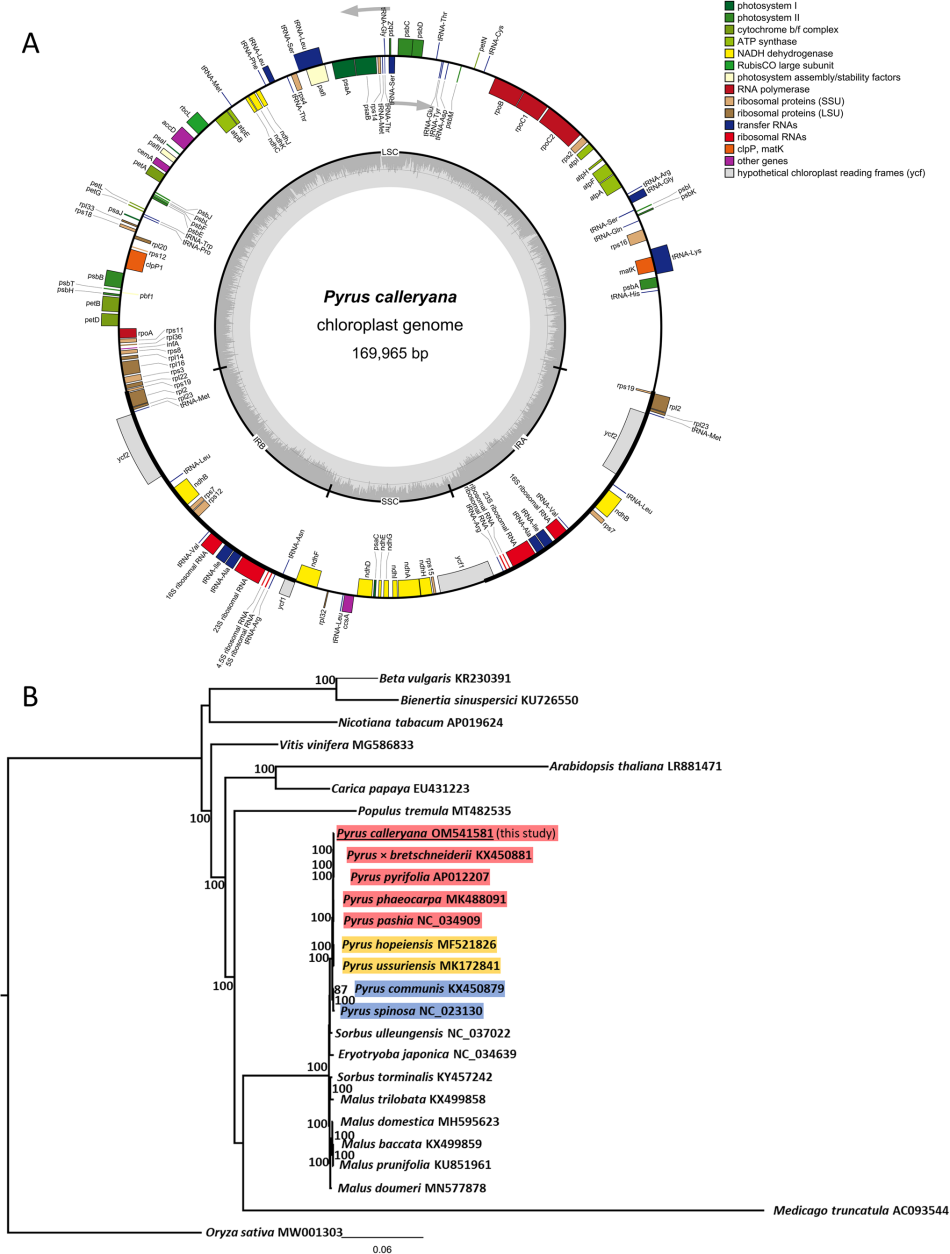
Chloroplast genome of *Pyrus calleryana*. **A** Genome map and annotation details of OM541581. Color legend on the right details the character or function of annotated features. Features placed on the inside of the outer ring are minus ( −) strand and those on the outside of the outer ring are plus ( +) strand; the grey arrows indicate the direction of translation. Long genomic segments are indicated: LSC — large single-copy region; SSC — small single copy region; IRA and IRB — genomic inverted repeat regions A and B, respectively. The inner circle depicts the GC content. **B** Phylogenetic placement of the newly developed chloroplast genome of *Pyrus calleryana*. All chloroplast genomes used for analysis are labeled with their respective GenBank IDs, and bootstrap support > 70% is identified. MAFFT-aligned chloroplast genomes were assessed for the nucleotide substitution model (GTR + G + I), corrected for parsimony and maximum likelihood, and bootstrapped 100 times. The reticulated tree was re-rooted using the single monocot species, *Oryza sativa*. For reference, the genetic distance legend is placed at the bottom. Colored nameplates denote the geographic origins of given *Pyrus* species: East Asia — red; Middle East and Russia — yellow; Europe and the Mediterranean Basin — blue

The online tool SSR Finder (https://ssr.nwisrl.ars.usda.gov/) was used to scan the retrieved chloroplast genome for perfect and imperfect microsatellites using the default permissive parameters. Comparisons with reference and among *P. calleryana* genomes were carried out using the online tool mVista Viewer (https://genome.lbl.gov/vista/mvista/submit.shtml) and investigated in detail for the conserved sequences reports. Analyses of the phylogenetic placement of the *P. calleryana* plastidic genome were based on the alignment with available plastidic genomes of other *Pyrus* spp., related rosids, asterids, Amaranthaceae, and the monocot *Oryza sativa* serving as the root. Entire plastidic genome sequences identified by their respective GenBank accession numbers in Fig. 1B were aligned using the automatic mode with default parameters in MAFFT v.7.487 (Kuraku et al. 2013). The resultant sequence matrix was analyzed for the best substitution model (GTR + G + I), whereas the resultant genetic distance matrix was corrected for parsimony and maximum likelihood, and bootstrapped 100 times using package phanghorn v.2.5.3 (Schliep et al. 2017) in R v.4.1. The reticulated tree was re-rooted using the single monocot species *Oryza sativa* in FigTree v.1.4.4 (Rambaut 2012).

## Results and discussion

Our approach resulted in a circular molecule of 159,965 bp in length with the overall coverage of 1014 × (minimum depth: 102 ×) and the GC content of 36.56%. Out of 24,759,507 paired-end reads in SRR16505594, 1,590,849 were used towards accretion of the genome. Execution of the code took less than 5 min on AMD Ryzen 9 5900X 12-cores machine; majority of that time was spent downloading the reads archive file. Classical quadripartite structure of *P. calleryana* chloroplast was recovered: two inverted repeats regions (IRA and IRB; each 26,392 bp) separated the large single-copy region (LSC; 87,942 bp) and the small single-copy region (SSC; 19,239 bp). In total, 125 unique features were annotated, whereas seven genes typically found in chloroplasts (*chlL*, *chlN*, *ycf15*, *trnP*-*GGG*, *psaM*, *ycf12*, *infA*) were absent both from the chloroplast genome of *P. calleryana* and from the reference. The annotated features included 83 protein coding genes, 38 tRNA coding genes, and 4 rRNA coding genes (Fig. 1A). The long genomic IRs showed almost identical content of annotated features in the same inverted order; the single difference was lack of tRNA-Asn in IRA (Fig. 1A). Exclusively the compound microsatellites were identified (*n* = 11), suggesting very limited potential of this analytical technique. Comparisons of the reference *P. phaeocarpa* and the *P. calleryana* chloroplast genomes indicated their high overall nucleotide identity of 99.79% (Suppl. File 2). The average nucleotide identity across 99 exons of 78,114 bp in total was 99.99%, with minor differences of up to 0.02% detected in only 10 exons. The 17 introns totalling 12,993 bp were on average identical at 99.77%, with differences of up to 3.1% in four introns. Relatively the lowest identity of 99.49% was identified across the 68 intergenic regions of total length 68,343 bp, with perfect matches in 33 introns and differences of up to 3.7%. This result underscores the relationship between both species as closer than inferred in other studies using more limited sequences (Zheng et al. 2014). This also validates our bioinformatic pipeline as usable at infrageneric levels and encourages its application to taxonomically close species.

To evaluate our bioinformatics approach, we applied the same pipeline on three other available genomic DNA datasets of *P. calleryana.* No major differences were apparent among the assembled four chloroplast genomes (Suppl. File 2). They formed an alignment of 160,362 bp with 159,722 invariable sites and 1484 single nucleotide polymorphisms overall across the four genomes, predominantly in the non-coding regions. The pairwise genetic distances recovered were smaller than 0.0004 (Nowicki et al., data not shown). The retrieved single-nucleotide polymorphisms were evaluated regarding their analytical use for restriction digest. Two such identified candidate polymorphisms proved successful when tested on our previous collection of *P. calleryana* DNA (Sapkota et al. 2021), giving rise to a haplotyping platform leveraged in our ongoing studies (Nowicki et al., data not shown). Furthermore, as reported in a previous study using four *P. calleryana* accessions (Zheng et al. 2014), the sequence analogous to the *trnL-F* was invariable among our four assembled genomes. But, unlike that study reporting identity in the *accD-psaI* intergenic region, all four genomes assembled here showed substantial nucleotide polymorphisms across the 150 bp located in the center of that locus (Suppl. File 3; Nowicki et al., data not shown). Collectively, these results underscore the high molecular variability detected in *P. calleryana* species complex using other approaches (Santamour and Demuth 1980; Sapkota et al. 2021, 2022).

Callery pear chloroplast placed among the other genomes of the related rosids, embedded together with other *Pyrus* spp. chloroplast genomes. Furthermore, in agreement with the three centers of origin for this genus, the chloroplast genomes of Asian pears *P. pyrifolia* (Terakami et al. 2012), *P.* × *bretschneiderii* (Cho et al. 2019), *P. phaeocarpa* (Xiang et al. 2019), and *P. pashia* (Bao et al. 2017) were in the immediate vicinity of the *P. calleryana*. The Middle-Eastern/Russian species *P. hopeiensis* (Li et al. 2018) and *P. ussuriensis* (Gil et al. 2019) placed between the Asian cluster and the European cluster of *P. communis* (Gil et al. 2019) and *P. spinosa* (Korotkova et al. 2018). That placement is in accord with other studies that investigated the *Pyrus* spp. phylogeny based on the plastidic sequences (*trnL-F*, *accD-psaI*) and the nuclear one *LFY2int2-N* (Zheng et al. 2014), or the sequence data of 702 microsatellite loci across *Pyrus* spp. (Jiang et al. 2019).

Analyses of the complete chloroplast genome sequences improved the phylogenetic resolution (bootstrap values) compared with those previous studies, similar to the genome-wide SNP-based phylogeny (Wu et al. 2018). As such, our chloroplast genome analyses reflect well the geographic distribution and evolutionary history of the genus. This resource is currently being leveraged in the follow-up studies of the invasive history of *P. calleryana* in the USA.

## Supplementary Information

Below is the link to the electronic supplementary material.

Supplementary file1 (TXT 0 KB)

Supplementary file2 (PDF 31 KB)

Supplementary file3 (PDF 49 KB)

## Data Availability

The annotated chloroplast genome sequence that supports the findings of this study is available at GenBank of NCBI: OM541581.1. The code, genome sequence, annotation, and Supplementary Files are additionally released by Dryad, under https://doi.org/10.5061/dryad.37pvmcvmq.
