# Supplementary material for: Chloroplast genome of the invasive Pyrus calleryana underscores the high molecular diversity of the species
Source: J Appl Genet. 2022 May 6;63(3):463–7. doi: 10.1007/s13353-022-00699-8 (PMC9365713; doi:10.1007/s13353-022-00699-8)
Supplement: Supplementary file 2 — Supplementary file2 (PDF 31 KB) [file 13353_2022_699_MOESM2_ESM.pdf]

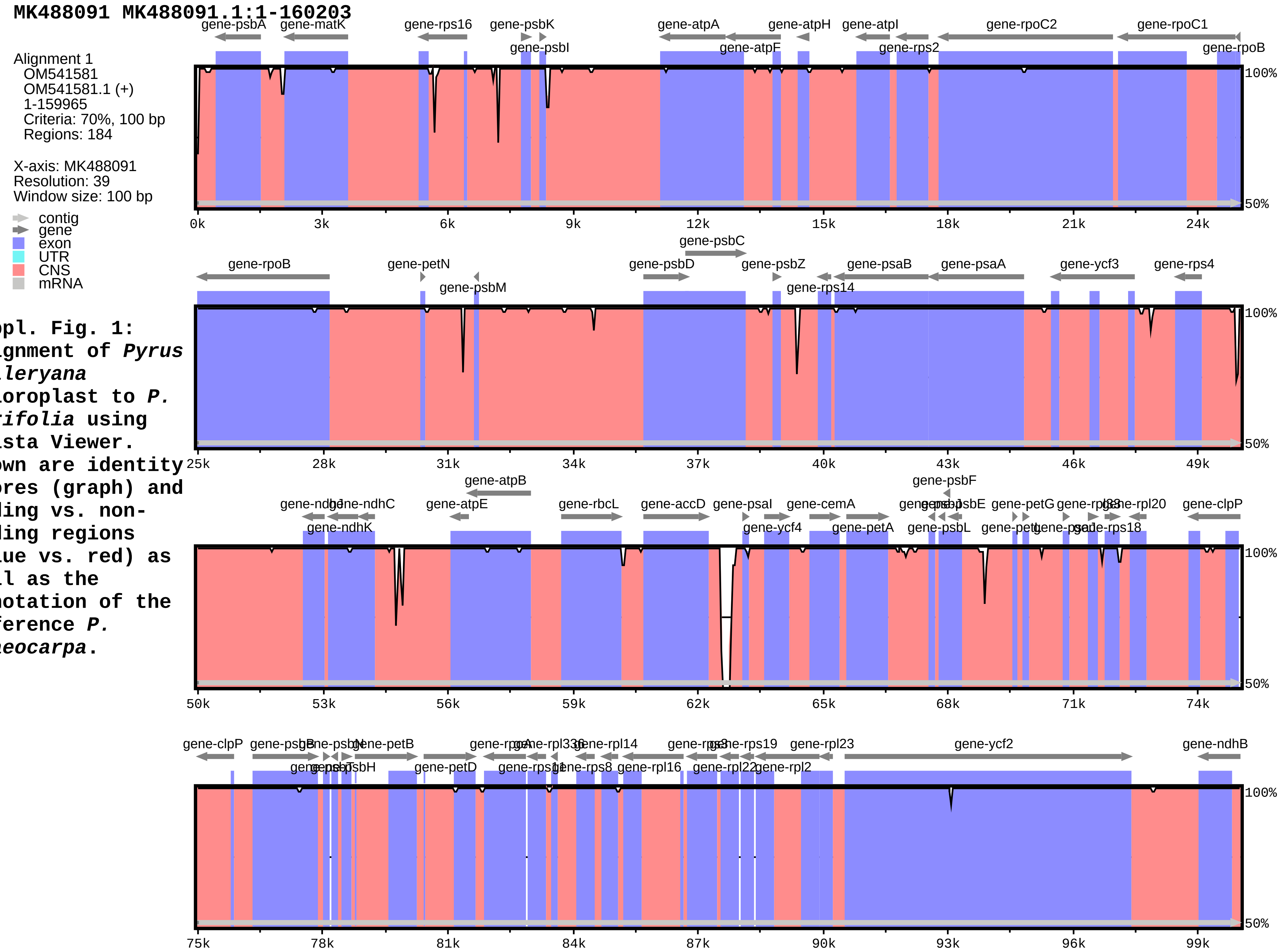

Suppl. Fig. 1: Alignment of *Pyrus calleryana* chloroplast to *P. pyrifolia* using mVista Viewer. Shown are identity scores (graph) and coding vs. non-coding regions (blue vs. red) as well as the annotation of the reference *P. phaeocarpa*.

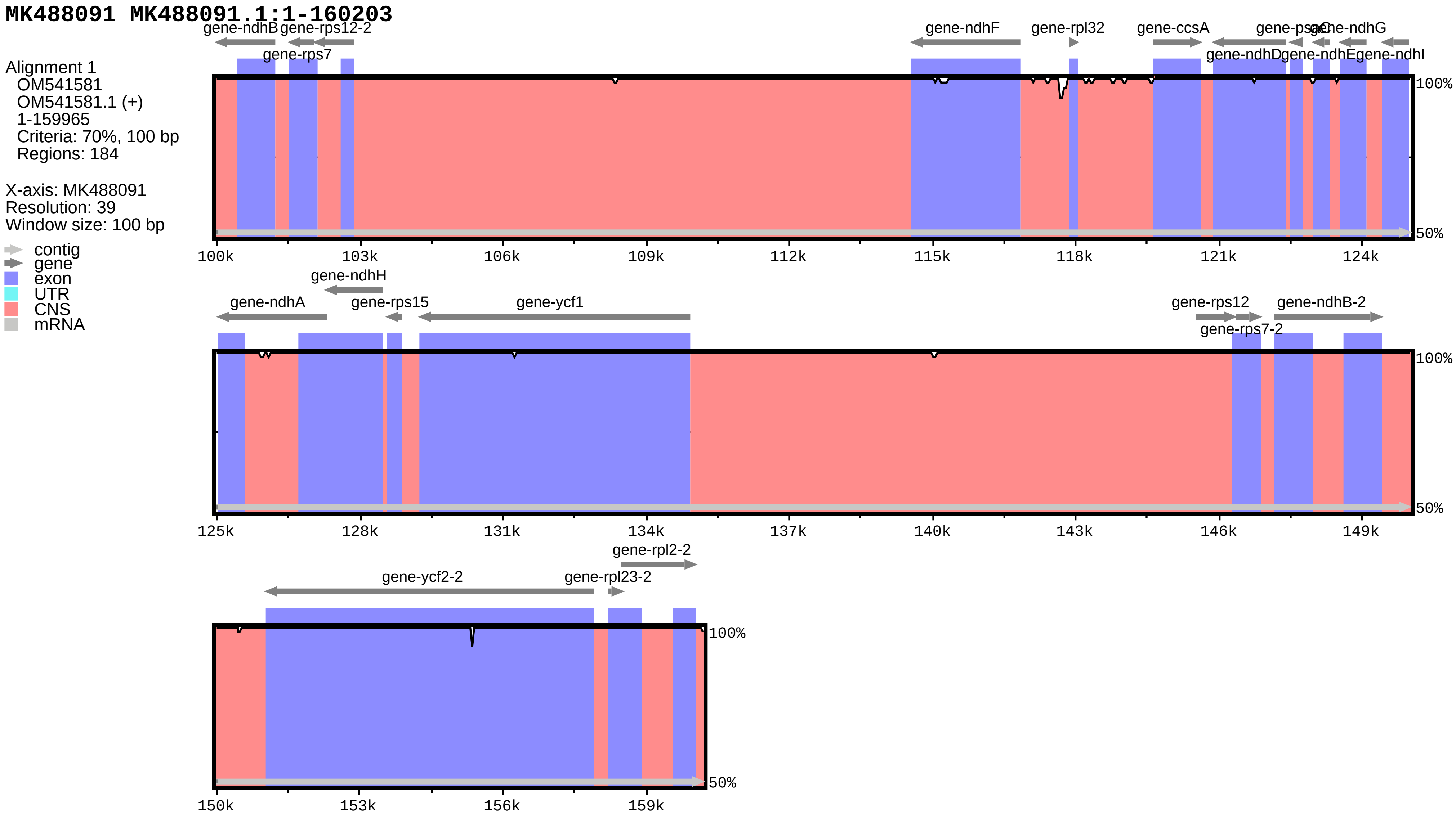
MK488091 MK488091.1:1-160203
Alignment 1
OM541581
OM541581.1 (+)
1-159965
Criteria: 70%, 100 bp
Regions: 184
X-axis: MK488091
Resolution: 39
Window size: 100 bp
contig
gene
exon
UTR
CNS
mRNA
gene-ndhB
gene-rps12-2
gene-rps7
gene-ndhF
gene-rpl32
gene-ccsA
gene-psaC
gene-ndhG
gene-ndhD
gene-ndhE
gene-ndhI
100%
50%
100k
103k
106k
109k
112k
115k
118k
121k
124k
gene-ndhH
gene-ndhA
gene-rps15
gene-ycf1
gene-rps12
gene-ndhB-2
gene-rps7-2
125k
128k
131k
134k
137k
140k
143k
146k
149k
gene-rpl2-2
gene-ycf2-2
gene-rpl23-2
150k
153k
156k
159k
