# Supplementary material for: Chloroplast genome of the invasive Pyrus calleryana underscores the high molecular diversity of the species
Source: J Appl Genet. 2022 May 6;63(3):463–7. doi: 10.1007/s13353-022-00699-8 (PMC9365713; doi:10.1007/s13353-022-00699-8)
Supplement: Supplementary file 3 — Supplementary file3 (PDF 49 KB) [file 13353_2022_699_MOESM3_ESM.pdf]

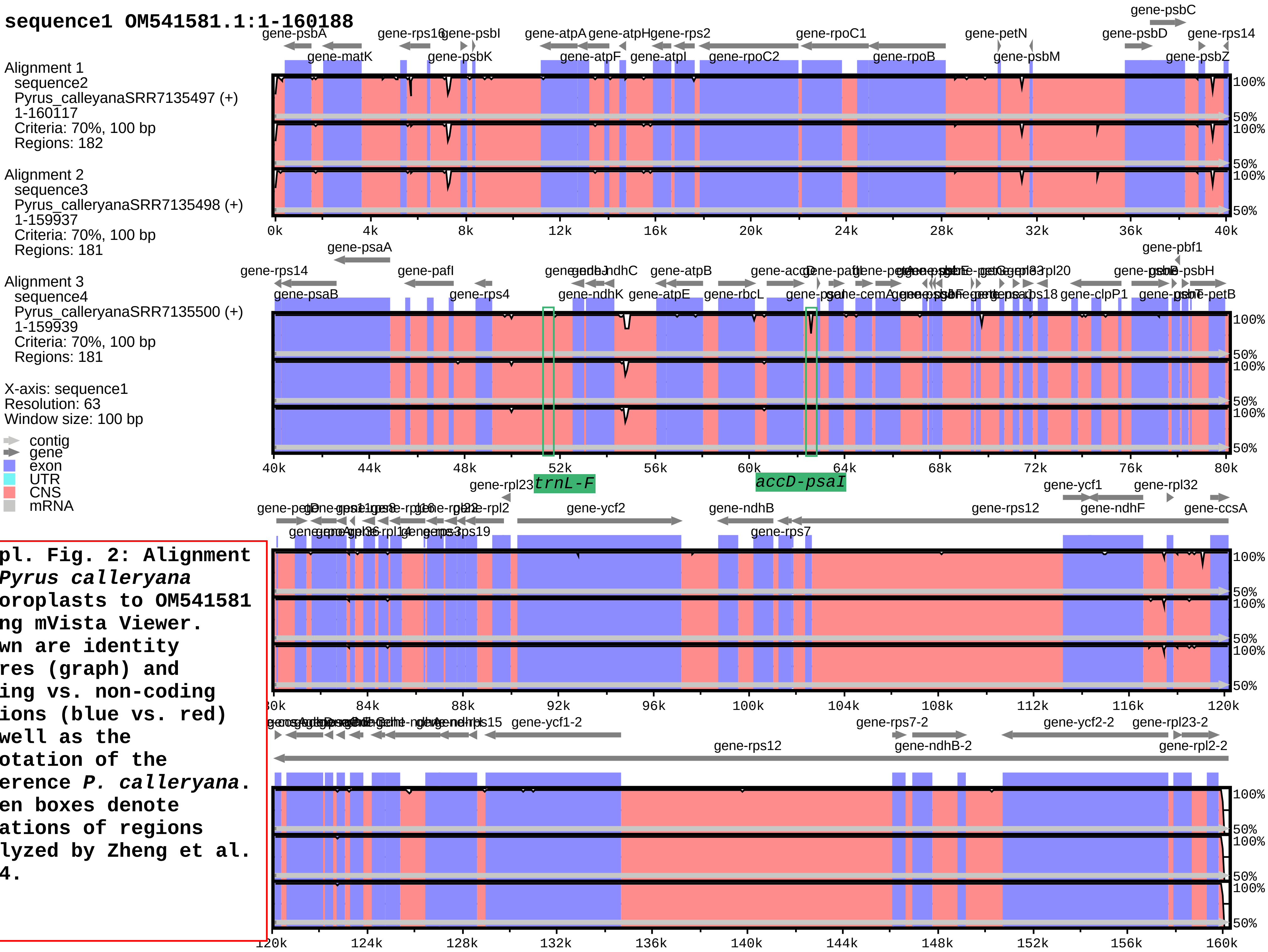

Suppl. Fig. 2: Alignment of *Pyrus calleryana* chloroplasts to OM541581 using mVista Viewer. Shown are identity scores (graph) and coding vs. non-coding regions (blue vs. red) as well as the annotation of the reference *P. calleryana*. Green boxes denote locations of regions analyzed by Zheng et al. 2014.
